# Fronto-Temporal Circuits in Musical Hallucinations: A PET-MR Case Study

**DOI:** 10.3389/fnhum.2018.00385

**Published:** 2018-09-27

**Authors:** Carlo Cavaliere, Mariachiara Longarzo, Mario Orsini, Marco Aiello, Dario Grossi

**Affiliations:** ^1^NAPLab, IRCCS SDN Istituto di Ricerca Diagnostica e Nucleare, Naples, Italy; ^2^Department of Psychology, University of Campania Luigi Vanvitelli, Naples, Italy

**Keywords:** PET/MR, musical hallucinations, neuropsychological assessment, glucose metabolism, fMRI, DTI

## Abstract

The aim of the study is to investigate morphofunctional circuits underlying musical hallucinations (MH) in a 72-years old female that underwent a simultaneous 18fluoredeoxyglucose positron emission tomography (PET) and advanced magnetic resonance (MR) exam. This represents a particular case of MH occurred in an healthy subject, not displaying neurological or psychopathological disorders, and studied simultaneously with a multimodal approach. For the resting-state fMRI analysis a seed to seed approach was chosen. For the task-based fMRI, 4 different auditory stimuli were presented. Imaging findings were compared with data obtained by ten healthy controls matched for age and sex. Neuropsychological evaluation and questionnaires investigating depression and anxiety were also administered. PET findings showed hypermetabolism of: superior temporal gyri, anterior cingulate, left orbital frontal, and medial temporal cortices. Structural MRI did not show macroscopical lesions except for gliotic spots along the uncinate fascicle pathways with an increased cortical thickness for the right orbitofrontal cortex (*p* = 0.003). DTI showed increased fractional anisotropy values in the left uncinate fascicle, when compared to controls (*p* = 0.04). Resting-state fMRI showed increased functional connectivity between the left inferior frontal gyrus and the left temporal fusiform cortex (*p* = 0.01). Task-based fMRI confirmed PET findings showing an increased activation of the superior temporal gyrus in all the auditory tasks except for the monotone stimulus, with a significant activation of the left orbital frontal cortex only during the song in foreign language, object of MH. Results on cognitive test did not show cognitive impairment, excepting for the performance on Frontal Assessment Battery where the patient fails in the cognitive domains of conceptualization, sensitive to interference, and inhibitory control. The subject did not show depressive or anxiety symptoms. Summarizing, multimodal imaging analyses in the MH case showed a microstructural alteration of the left uncinate fascicle paralleled by an increased metabolism and functional connectivity of cortical regions that receive left uncinate projections (orbital frontal cortex, and medial temporal cortex). This alteration of fronto-hyppocampal circuits could be responsible of retrieval of known songs even in the absence of real stimuli.

## Introduction

No studies have investigated the neural bases of musical hallucinations (MH) and their neuropsychological correlates. The present study novelty consists in comprehensive investigation of MH manifestation in a subject not displaying any of the typical syndromes or impairments subtending MH. A multimodal approach including several neuroimaging techniques and global cognitive evaluation was performed in this case study.

### Case Description

A 72-years old female was referred to the IRCCS SDN with medical prescription to undergo a Magnetic Resonance Imaging with simultaneous Positron Emission Tomography (MRI-PET) exam in order to investigate whether the MH she referred had an epileptic nature. She has 5 years education and she is housewife; she does not display neurological illnesses, neurodegenerative disorders, or psychopathological conditions. The onset of MH was sudden, six months since neurological evaluation, coinciding with a period of stressful events affecting the health of her husband. From the onset, her MH present daily, mainly during the afternoon.

From the clinical interview made by the neuropsychologist, emerged that familiar Neapolitan, English, and French songs (3–4 current songs with no special preference, accompanied with music, without amplitude alterations neither memories/emotions associated) characterize patients’ MH that do not interfere with the sleep and with daily activities and are slightly weakened by listening radio or watching television. The patient always hears the same songs, in particular singers’ original voice without instrumental contribution. The patient did not report co-existing auditory hallucinations.

She also experienced hearing impairment assessed by an otolaryngologist who diagnosed presbyacusys and recommended the use of bilateral mobile acoustic prostheses.

The present study was carried out in accordance with the Declaration of Helsinki and the local ethics committee approved it. Written informed consent was obtained by the subject, also specifically for the publication of collected data, in anonymous form, in the present case report.

### Cognitive Evaluation

In order to examine the patients’ clinical and cognitive profiles the following battery of tests was administered. The Beck Depression Inventory-II (BDI-II; Beck et al., 1996) used to assess depression, higher scores indicate more severe depressive symptoms; the State-Trait Anxiety Inventory (STAI Y1 e Y2; Spielberger et al., 1983) used to explore the presence of anxiety, the threshold value predictive of anxiety symptoms is set at a score of 40; the Mini Mental State Examination (MMSE; Magni et al., 1996) for global cognitive screening; the Frontal Assessment Battery (FAB; Apollonio et al., 2005) is sensitive to specific frontal cognitive functions degeneration; the phonologic fluency (Novelli et al., 1986), the semantic fluency (Spinnler and Tognoni, 1987) and the Raven’s Colored Progressive Matrices (Basso et al., 1987) investigate impairment in executive functions; verbal span (Wechsler, 1955) investigates memory function.

### PET/MR Acquisition Protocol

PET/MR data were simultaneously acquired using a Biograph mMR tomograph (Siemens Healthcare, Erlangen, Germany) designed with a multi-ring LSO detector block embedded into a 3 T magnetic resonance scanner. The PET procedure was in compliance with the European Association of Nuclear Medicine procedure guidelines for brain PET imaging (Varrone et al., 2009). Accordingly, the subject rested in a quiet and warm dark room for 15 min before FDG administration and during the uptake period. Brain FDG-PET was acquired for 15 min after the patient rested for 15 min in a quiet dark room before 250 MBq [18F]-FDG dose administration and during the uptake period (20–25 min). Subject was not allowed to consume any food or sugar for at least 6 h prior to FDG injection. Blood glucose was measured at arrival at the PET center and FDG was injected only if glycaemia was below 120 mg/dl. The PET data were acquired in sinogram mode for 15 min; matrix size was 256 × 256. PET emission data were reconstructed with ordered subset-expectation maximization (OSEM) algorithm (21 subsets, 4 iterations) and post-filtered with a three-dimensional isotropic gaussian of 4 mm at FWHM, resulting in a final spatial resolution of approximately 6 mm along each direction. Attenuation correction was performed using MR-based attenuation maps derived from a dual echo (TE = 1.23–2.46 ms) Dixon-based sequence (repetition time 3.60 ms), allowing for reconstruction of fat-only, water-only, and of fat–water images. The resulting segmentation of background, fat, and muscles is used to estimate head profiles needed for calculation of attenuation (Martinez-Möller et al., 2009; Aiello et al., 2015).

During PET acquisition, the following MRI sequences were run sequentially:

(i) Three-dimensional T1-weighted magnetization-prepared rapid acquisition gradient-echo sequence (MPRAGE, 240 sagittal planes, 214 × 214 mm^2^ field of view, voxel size 0.8×0.8×0.8 mm^3^, matrix TR/TE/TI 2400/2.25/1000 ms, flip angle 8°, TA = 6′18′′)
(ii) T2^∗^-weighted single-shot EPI sequence (voxel-size 4×4×4 mm^3^, TR/TE = 1000/21.4 ms, flip angle 82°, 350 time points, FOV read = 256, distance factor = 0, TA = 5′56′′) for rs-fMRI.
(iii) T2^∗^-weighted single-shot EPI sequence BOLD (voxel size 4×4×4 mm^3^, TR/TE = 1000/21.4 ms, flip angle = 82°, 240 time points, FOV read = 256, distance factor = 0, TA = 4′00′′) for task-related fMRI.
(iv) T2^∗^-weighted single-shot EPI sequence (voxel-size 2×2×2 mm^3^, TR/TE/ = 3851/84.2 ms, flip angle 90°, FOV = 256x256 mm^2^, b max value = 1500, b0 number = 8) for DTI.

Additionally, axial T2-FLAIR and 3D T2-TSE were also acquired to exclude brain lesions.

Ten age-matched female healthy volunteers (HCs) (70 ± 3 year, age range 67–74) underwent the same MR acquisition protocol, except for the task-fMRI. The local ethical committee’s approval and informed written consent were obtained before the study.

### Task fMRI-BOLD

Four BOLD conditions were acquired, set in blocked design, each composed of two paradigms. The first paradigm was a stimulation, the second one was the rest; each paradigm had duration of 24′′. Every BOLD sequence was composed of 5 blocks for a total duration of 4 min. For each condition a different stimulus was used, consisting in refrain of songs or beep sound:

• *1: native language song included in patient’s hallucinations*
• *2: foreign language song included in patient’s hallucinations*
• *3: foreign language song unknown to the patient*
• *4: a high frequency monotone beep sound*

The stimulus for the fMRI-BOLD sequence was administered by means of standard MRI headphones, and a loudspeaker placed under the patient’s crib.

Motion correction to BOLD sequences has been applied to eliminate involuntary patient’s head motions. The anatomical sequence T1 MPRAGE has been co-registered and it has been used as base of the statistical parametric maps. Sequence T2^∗^-weighted to distortion correction was used to uniform the field’s inhomogeneity.

### Data Pre-processing

#### Structural MR Imaging

The FreeSurfer (v5.1) toolkit^1^ (Dale et al., 1999) was used to process anatomical MPRAGE images from the subjects to construct white matter, pial, inflated, and spherical brain surfaces. Briefly, this processing includes spatial inhomogeneity correction, non-linear noise-reduction, skull-stripping, subcortical segmentation, intensity normalization, surface generation, topology correction, surface inflation, registration to a spherical atlas and thickness calculation (Fischl and Dale, 2000). This approach matches morphologically homologous cortical areas based on the cortical folding patterns with minimal metric distortion and allows sampling at subvoxel resolution and detection of cortical thickness differences at the sub-millimeter level. Cortical thickness was estimated at each point across the cortical mantle by calculating the distance between the gray/white matter boundary and the cortical surface.

### Statistical Analysis

White matter hyperintensities of presumed vascular origin (WMH), are divided into periventricular (PWMH) and deep (DWMH) and assessed by a visual rating scale (Fazekas score), commonly used in clinical settings (Fazekas et al., 1987). For the statistical analysis of cortical thickness, we chose a Bayesian approach that is thought to be prudent, preventing the overestimation of evidence in favor of an effect (Molino et al., 2014). We thus used a Bayesian inferential method (SingleBayes.exe) (Crawford and Garthwaite, 2007) that allows testing if the patient’s values are significantly above (or below) the respective values of a small control sample.

### Results

#### Cognitive Results

Subjects’ scores on clinical questionnaires and cognitive tests are summarized in **Table 1**.

**Table 1:** Patients’ scores on clinical questionnaires and rough (and adjusted) scores on cognitive tests.

Clinical questionnaires	Rough (and adjusted) score	Cut-off
BDI-II	8	No dep. 0–13 Mild Dep. 14–19 Mod. Dep. 20–29 Severe Dep. 30–63
STAI Y1	36	≤40
STAI Y2	41	≤40
***Cognitive tests***		
MMSE	29 (28.3)	>24
FAB	10 (11.3)^∗^	>13.48
Phonologic Fluency	24 (32.6)	>17
Semantic Fluency	65 (73)	>33.25
Raven Progressive Matrices	18 (22.5)	>18.9
Digit Span	7 (7.5)	>3.75

*BDI-II, Beck Depression Inventory-II; *STAI Y1 and Y2*, State-Trait Anxiety Inventory; *MMSE,* Mini Mental State Examination; *FAB,* Frontal Assessment Battery. ^∗^Impaired performance*.

The patient did not show depressive symptoms and state anxiety symptoms, just the score on trait anxiety questionnaire slightly exceeds the cut-off. Results on cognitive test did not show cognitive impairment, excepting for the performance on the FAB where abilities in the tests of similarities (conceptualization), conflicting instructions (sensitive to interference), and Go-Nogo (inhibitory control) were compromised.

#### Imaging Findings in MH

**FIGURE 1:**
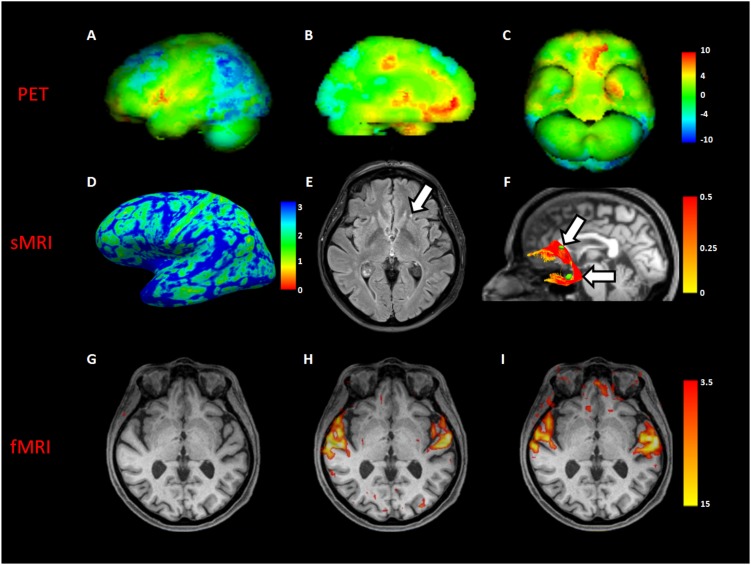
Neuroimaging analyses in MH case. The first row showed a volumetric representation of PET results in MH, compared to a dataset of age-matched controls **(A)**, left lateral view; **(B)**, left medial view; **(C)**, inferior view of brain, respectively). The second row showed both macro- and micro- structural MRI (sMRI) results in the MH case; in **(D)**, a left view of cortical thickness map in millimeters; in **(E)**, an axial slice of fluid attenuation inversion recovery (FLAIR) sequence showing a gliotic spot (white arrow) in the left white matter frontal region; in **(F)**, the reconstruction of the left uncinate fascicle represented in fractional anisotropy scalar values (white arrows indicate gliotic spots segmented in green). The third row showed an axial slice with superimposed functional MRI (fMRI) activation during beep **(G)**, unknown song **(H)**, and known song in foreign language recognized as object of MH **(I)**.

Comparing MH case with a standardized PET database matched for age, a significant increased glucose metabolism was detected in superior temporal gyri, anterior cingulate, left orbital frontal, and left medial temporal cortices. Moreover, a significant decreased metabolism was found in the bilateral frontoparietal and parietooccipital cortices (**Figures 1A–C**).

Structural MRI of did not show macroscopical lesions except for subcortical gliotic alterations in the bilateral frontal and temporal white matter, along the pathways of the uncinate fascicle (Fazekas score = 1 (periventricular white matter) +1 (deep white matter)). Cortical thickness analysis showed a significant increase in the right lateral orbitofrontal cortex (MH: 2.75, CTRs: 2.51 ± 0.05; *p* = 0.003) (**Figures 1D,E**).

DTI showed a close spatial relationship between gliotic alterations and the pathway of both the uncinate fascicles, with a significant increase of the mean tract fractional anisotropy of the MH left uncinate fascicle, when compared to controls (0.44 vs. 0.35 ± 0.04 in HCs; *p* = 0.04) (**Figure 1F**).

Resting-state fMRI showed increased functional connectivity between the left inferior frontal gyrus and the left temporal fusiform cortex (*p* = 0.01). Task-based fMRI confirmed PET findings showing an increased activation of the superior temporal gyrus in all the auditory tasks, excepting for the “beep” stimulus not perceived by the patients because of her presbyacusys (**Figures 1G–I**). Moreover, a significant activation of the left orbital frontal cortex only during the song object of MH, recognized during imaging session, was found (**Figure 1I**).

## Background

Musical hallucinations represent a quite rare phenomenon in which subjects perceive music/melodies/songs in the absence of an external auditory source. Such perceptual experience is presented as intrusive and independent of the subjective control, seems to be more common in women and has been first documented in patients by Rozanski and Rosen (1952) and Berrios (1990).

Converging evidence by literature about MH recognize their composite nature and heterogeneous neuroanatomical bases. Bernardini et al. (2016) reported that, on subjects showing MH, several neuroimaging procedures have been used singularly, i.e., PET, single photon emission computed tomography (SPECT), magnetoencephalography (MEG) and functional magnetic resonance imaging (fMRI), producing several data about the association between MH and neuroimaging findings. According to what emerged by previous studies, the superior temporal sulcus is the brain area mainly involved in this phenomenon, because of its activation in response to musical stimuli; additional cortical and subcortical regions such as orbitofrontal cortex, basal ganglia, and precuneus seem to participate in MH experience (Bernardini et al., 2016). For example, Kumar et al. (2014) by using MEG, found orbitofrontal cortex (OFC) activation during MH and hypothesized its role in the reproduction of musical memories, on the basis of OFC involvement in assigning the emotional value of stimuli (Rolls, 2007) and on the basis of the correlation between its activity and distress caused by phantom percept of tinnitus (Joos et al., 2012).

Musical hallucinations are reported to be associated with neurological impairments (both in brain lesions and in neurodegenerative diseases), psychiatric disorders (e.g., schizophrenia), hearing impairment, epilepsy, and intoxication/pharmacology (Evers and Ellger, 2004).

About hearing loss, according to interpretation Griffiths (2000), the lack of afferent stimuli to the auditory processing induces a spontaneous production in the auditory network, similar to what happens in the Charles’ Bonnet Syndrome (Burke, 2002).

Golden and Josephs (2015) provided the largest series of MH cases divided in neurologic, psychiatric, structural lesions, drug toxicity or withdrawal, and not otherwise classifiable group including patients with different co-morbidities and symptoms.

Therefore, to date and to our knowledge, no studies examined MH in healthy subjects, or with a multimodal imaging approach, neither with an accurate clinical and cognitive assessment.

In the present study we aimed to investigate neuroimaging findings and cognitive performance of a female subject whose only symptom is MH phenomenon, consisting in repetition, of familiar songs. We used a multimodal neuroimaging approach by combining simultaneous PET and functional MRI acquisitions and we expected that, if organic causes are excluded, an alteration of fronto-hyppocampal circuits might occur, responsible of retrieval of known songs even in the absence of real stimuli.

## Discussion

MH represents a quite rare phenomenon, with heterogeneous neuroanatomical bases, often related to neurological, psychiatric, or oncological diseases.

Here we describe a case of MH occurred in a healthy 72 y-o female subject, studied with clinical and cognitive assessment and by simultaneous PET/MR imaging.

The patient showed a normal cognitive profile, with no signs of impairment excepting for the performance on three selective FAB subtests. In particular, she failed in the similarities, conflicting instructions and Go-Nogo tasks, all managed by the frontal lobe, mainly the orbitofrontal cortex that regulates superior intellectual functions (Rolls, 2004). During the interview with the subject it emerged that she is aware about the absence of the musical stimuli in the external world and that nobody else heard the music, hypothesizing about the subjective generation of songs. The consciousness about the self-generation of auditory contents distinguishes hallucinations from hallucinosis, which implies the self-attribution of perceived stimuli. In the present case it would be more correct referring to musical hallucinosis phenomenon, rather than musical hallucinations, according to Vanneste et al. (2013).

Cognitive functions in patients presenting MH have been poorly investigated, most of the available data about the relationships between cognitive functions and hallucinations come from researches on patients with neurodegenerative disorders (Ruiz-Almazán et al., 2009). Grossi et al. (2005) found impaired performances in frontal sensitive tasks in non-demented patients with Parkinson disease presenting auditory hallucinations; such deficit was attributed to dysfunction in controlling and monitoring functions sustained by prefrontal areas. Some studies reported that mild cognitive impairment and hearing loss could be predictor factors of MH (Paquier et al., 1992; Stephane et al., 1999; Ruiz-Almazán et al., 2009) and recently a parallel has been made between MH and tinnitus (equally perceived in the absence of external sources) in which a top-down executive control deficit seems to occur (Araneda et al., 2015).

In the present study, the multimodal approach adopted highlights several intriguing neuroimaging findings. Compared to a control dataset matched for age and sex, PET data show two opposite patterns of brain metabolism in MH subject. Hypometabolism occurred in bilateral frontoparietal cortices, included in the extrinsic control network for the perception of the external stimuli (Corbetta and Shulman, 2002) (see below). Conversely, we found hypermetabolism in left orbitofrontal and temporomedial cortices, anterior cingulate and superior temporal gyrus bilaterally, that has been reported as crucial in MH, receiving primary outside auditory inputs. In our case, the increased glucose metabolism in this area is due to continuous MH reported by subject during the glucose uptake period. The hypermetabolism occurred in left orbitomedial and bilateral cingulate regions, suggests an altered monitoring and processing of reality and the conscious attribution of a self-generated “stimulus” to the external word. Furthermore, left medial temporal cortices hypermetabolism implies memory activations related to superior temporal gyri devoted to song processing, this could result in memory activation of familiar songs.

All of these regions are projection territories of the uncinate fascicle (Catani et al., 2002) dealing with emotions and episodic memory (Von Der Heide et al., 2013). Here, macroscopic MRI evaluation showed subcortical gliotic alterations along bilateral uncinate fascicles pathways with a significant increase of FA only in the left tract. Other studies demonstrated higher FA values of the arcuate fascicle in schizophrenic patients with auditory hallucinations (Hubl et al., 2004; Kubicki et al., 2005). Such finding, as in our case for the uncinate fascicle, could sustain an over activation of cortical regions associated with hallucinations (Kubicki et al., 2005). It is noteworthy, rs-fMRI showed increased functional connectivity between the left inferior frontal gyrus and the left temporal cortex, regions also detected as hypermetabolic to PET exam, and receiving the projections of the two branches (frontal and temporal) of uncinate fascicle. We hypothesized that in normal hearing individuals the capability in perceiving environmental auditory stimuli efficiently suppresses internal perception, whereas subjects with presbyacusis has a significant reduction in perceiving external auditory stimuli (as demonstrated in our case by the PET hypometabolism of extrinsic control network areas) and, as a result, internal perception can easily prevail. The insight perception are guided by emotional and cognitive subjects’ memories (in this case in the form of song with emotional value for our subject), recalled by hippocampus and sent to orbitofrontal cortex through the uncinate fascicle. Since the orbitofrontal hyperactivation, the subject could not be able in distinguishing from real (external) and unreal (internal) auditory stimuli, causing the subject to feel what is not in the environment. Such alteration could be attributable to a disconnection between the extrinsic network and the default-mode network. Several studies have reported the competing character of the two systems (Tian et al., 2007), and also that high prestimulus baseline activity in the intrinsic system, like that elicited by continuous MH, is associated with a tendency to ignore environmental stimuli (Sapir et al., 2005), like highlighted by PET hypometabolism in our case. Because very few persons with acquired deafness show this phenomenon it seems possible that the scare damage of the uncinate fascicle had a role in generating musical hallucinosis, misleading information between temporal, and orbitofrontal cortices therefore contributing to altered reality monitoring.

Our interpretative hypothesis appears strengthened by additional imaging findings we obtained by other techniques. In MH subject, we observed increased cortical thickness of the orbitofrontal cortex and rs-fMRI revealed enhanced connectivity between inferior frontal gyrus and temporal cortex. Moreover, fMRI performed during auditory task showed different results depending on the musical stimulus heard by the MH subject, except for a comparable activation of superior temporal gyri. The beep stimulus was not perceived, maybe due to the presbyacusis that affects superior temporal gyrus processing of auditory stimuli; during “O’ sole mio” song (one of the hallucination contents not recognized by the subject) and during “Happy” song (unknown to the subject) no brain activation was observed compared to resting-state condition; during “La vie en rose” (one of the hallucination contents recognized by the subject) significant hyperactivation of the left orbitofrontal cortex was detected.

Furthermore, functional imaging demonstrated hypermetabolism of the left medial temporal cortices by PET, and an increased functional connectivity of the left hippocampus during resting-state fMRI.

The fine definition of the neuroanatomical bases of MH, mainly in absence of an organic disease, could address for targets non-invasive treatments such as transcranial magnetic stimulation (TMS), tools that have already demonstrated their efficacy in patient symptoms relief (Cosentino et al., 2010).

## Concluding Remarks

Taken together, our results suggest a crucial involvement of prefrontal lobe in MH, both in cognitive and aspects neurofunctional perspectives. In sum, if on one hand the impaired auditory perception causes dysfunction of superior temporal gyrus, on the other one the retrieval of known songs from medial temporal cortices is misinterpreted by prefrontal cortices due to an alteration of uncinate fascicle, determining musical perception as real in the absence of external stimuli. We can infer that altered relationships between structures and network analyzed could have a crucial role in MH appearance and management.

## Ethics Statement

The present study was carried out in accordance with the Declaration of Helsinki. The protocol was approved by the IRCCS Pascale Ethics Committee. Written informed consent was obtained by the subject.

## Author Contributions

CC performed tractograpghy analysis and interpretation and wrote the paper together with ML who performed neuropsychological data collection, analysis and interpretation. MO performed fMRI bold paradigm and MRI acquisition and post processing. DG and MA developed the study concept and design and manuscript revision.

## Conflict of Interest Statement

The authors declare that the research was conducted in the absence of any commercial or financial relationships that could be construed as a potential conflict of interest.

## Footnotes

1 http://surfer.nmr.mgh.harvard.edu/
